# How to do it: Teaching surgical skills to medical undergraduates

**DOI:** 10.1016/j.amsu.2022.104617

**Published:** 2022-09-21

**Authors:** Victor Hugo Lara Cardoso de Sá, Giovanna Savoy Pazin, Pablo Eduardo Elias, Eduardo Achar, Gerson Vilhena Pereira Filho

**Affiliations:** a Plastic Surgery Department of ABC Medical School, University of São Caetano do Sul (USCS) Medical School, 50 Santo Antonio Street, São Caetano do Sul, SP, Brazil; b Surgical Abilities Module of USCS Medical School, University of São Caetano do Sul (USCS) Medical School, 50 Santo Antonio Street, São Caetano do Sul, SP, Brazil; c General Surgery Resident at PUC-Sorocaba Medical School, University of São Caetano do Sul (USCS) Medical School, 50 Santo Antonio Street, São Caetano do Sul, SP, Brazil; d Surgical Abilities Module of UNICID Medical School, University of São Caetano do Sul (USCS) Medical School, 50 Santo Antonio Street, São Caetano do Sul, SP, Brazil

**Keywords:** Education, Medical, Surgery, High fidelity simulation training, Undergraduate

## Abstract

Medical students must be capable of performing clinical and surgical procedures in outpatient care and initial emergency care in all stages of the biological cycle. Here, we describe the surgical skills schedule with different animal models fulfilled at the Municipal University of São Caetano do Sul (USCS) Medical School, São Caetano do Sul, SP, Brazil, during the surgical abilities module.

We retrospectively reviewed the surgical abilities module schedule provided at the USCS Medical School from 2015 until 2020; in this paper, we describe the use of different animal models. The activities were developed for two semesters during medical school and included an ox tongue, cylindrical Styrofoam, chicken leg and neck, live rabbits, and pigs.

Practical surgical teaching starts with sutures using the ox tongue, after which students are taught to perform tenorrhaphy using cylindrical Styrofoam and chicken legs, followed by vascular anastomosis using the chicken trachea and esophagus. Rabbits are appropriate for training a variety of procedures such as cystostomy, gastrostomy, and appendectomy. Pigs allow for the simulation of several types of procedures such as chest drainage.

Surgical training for medical undergraduates was demonstrated with an evolutionary intent, starting with simple sutures and ending up with basic emergency room surgical procedures.

## Highlights

• There is no standard of how to teach surgical skills for medical undergraduates.
• Many articles have been presented discussing different materials.
• Surgical skills should be taught in an evolutionary way – from simple to complex.
• We start simple and finish with surgical procedures in live animals.

## 1 Introduction

National and international guidelines define some mandatory procedures that must be taught to medical students such as suture techniques, thoracentesis, paracentesis, and pleural drainage. Students must be capable of performing clinical and surgical procedures in outpatient care and initial emergency care in all stages of the biological cycle [[1], [2], [3], [4]].

Surgical teaching around the world has been redesigned due to regulatory standards that restrict the use of animals. The replacement of dogs by pigs and dummies, among other simulation equipment, has greatly increased the costs of surgical technique training. Its direct consequence was the emergence of countless inanimate bench models for pig legs, chicken legs, and synthetic skin pads. Some advantages and disadvantages of different models have been summarized by Reznick et al. [3, [5], [6], [7], [8]].

Despite the teaching surgical panorama described above, animal models still play an important role in surgical simulation, as they seem to enable the development of better and more reliable technical skills, as well as the decision-making process, in a safer environment and with less pressure when compared to the actual scenario [9].

The aim of this paper is to describe the surgical skills schedule with different animal models fulfilled at the Municipal University of São Caetano do Sul (USCS) Medical School, São Caetano do Sul, SP, Brazil, during the surgical abilities module.

## 2 Material and methods

We retrospectively reviewed the surgical abilities module schedule implemented in the USCS Medical School from 2015 to 2020 and describe the use of different animal models to fulfill the objective of promoting the development of surgical skills in medical undergraduates.

## 3 Results

The activities were developed for two semesters, the sixth and seventh, during medical school. There were 15 activities in each semester, with 1 h and 30 min per week, totaling 45 h dedicated to practical surgical training. Our surgical team of professors included three plastic surgeons, one veterinary technician, and one laboratory technician.

Surgical training starts with theoretical guidance and video demonstrations of each procedure to be taught. At this time, the following topics were discussed: procedure indications, different possibilities of sutures and threads, and anesthesia. After the explanation, the students went to the lab to start the practice.

All the suture videos are available online at: https://www.youtube.com/channel/UC4WC5m9OBn7KdYuew077ZIw.

The first semester of training (see Table 1) was entitled “Basic Surgical Skills (BSSK)” as the students were supposed to learn basic surgical movements, posture, and how to manage surgical instruments, starting with a simple suture up to their first surgery on live animals. During BSS, students were seated at hexagonal tables with their individual materials: wooden plate, straight scissors, Mayo-Hegar needle holder, rat tooth forceps, appropriate suture thread, and the animal material to be used (Table 1). During all surgical activities, the teachers supervised the students. Typically, 10 students are assisted by each teacher.

**Table 1:** Sixth semester schedule – Basic Surgical Skills.

Week	Material	Activity	Group Dinamic
Week 1	Ox tongue	Simple suture	Individual activity
Week 2	Ox tongue	Donatti suture	Individual activity
Week 3	Ox tongue	Simple continuous suture	Individual activity
Week 4	Ox tongue	Anchored continuous suture	Individual activity
Week 5	Ox tongue	Inverted simple suture	Individual activity
Week 6	Ox tongue	Intradermic continuous suture	Individual activity
Week 7	Ox tongue	X suture	Individual activity
Week 8	Ox tongue	Skin graft and V–Y Flap	Individual activity
Week 9	Ox tongue	Suture revision	Individual activity
Week 10	Surgical instruments	Hand antisepsis, Surgical vestment and Operating table assembly	Individual activity
Week 11	Chicken foot + styrofoam	Kessler tenorraphy suture	Individual activity
Week 12	Chicken neck	Simple suture vascular anastomosis	Individual activity
Week 13	Chicken neck	Continuous suture vascular anastomosis	Individual activity
Week 14	Rabbits	Exploratory Laparotomy Cricothyroidostomy and Tracheostomy	Group activity (4 students per animal)
Week 15	Rabbits	Exploratory Laparotomy Cricothyroidostomy and Tracheostomy	Group activity (4 students per animal)

The second semester of training (see Table 2) was entitled “Advanced Surgical Skills (ASS)” (Table 2), as the students were supposed to review previous topics and evolve their surgical skills, becoming able to perform small surgical procedures on live animals. When practicing ASS, the students were seated at surgical tables (Picture 1), with the animals submitted to general anesthesia, in groups of four students per animal. During all surgical activities, teachers were available to supervise the students in the same proportion as previously mentioned. Animal use followed all Brazilian and international regulations [10].

**Table 2:** Seventh semester schedule – Advanced Surgical Skills.

Week	Material/Animal	Procedure	Grupo/Dinâmica
Week 1	Ox tongue	Sutures Review	Individual activity
Week 2	Chicken neck	Anastomosis Review	Individual activity
Week 3	Chicken foot + Styrofoam	Tenorrhaphy review	Individual activity
Week 4	Rabbits	Appendectomy	Group activity (4 students per animal)
Week 5	Rabbits	Appendectomy	Group activity (4 students per animal)
Week 6	Rabbits	Cystostomy	Group activity (4 students per animal)
Week 7	Rabbits	Cystostomy	Group activity (4 students per animal)
Week 8	Rabbits	Gastrostomy	Group activity (4 students per animal)
Week 9	Rabbits	Gastrostomy	Group activity (4 students per animal)
Week 10	Rabbits	Colostomy	Group activity (4 students per animal)
Week 11	Rabbits	Colostomy	Group activity (4 students per animal)
Week 12	Pigs	ATLS practical lesson	Group activity (4 students per animal)
		Chest drainage	
		Peritoneal lavage	
Week 13	Pigs	ATLS practical lesson	Group activity (4 students per animal)
		Chest drainage	
		Peritoneal lavage	
Week 14	Pigs	ATLS practical lesson	Group activity (4 students per animal)
		Chest drainage	
		Peritoneal lavage	
Week 15	Pigs	ATLS practical lesson	Group activity (4 students per animal)
		Chest drainage	
		Peritoneal lavage

**Picture 1:**
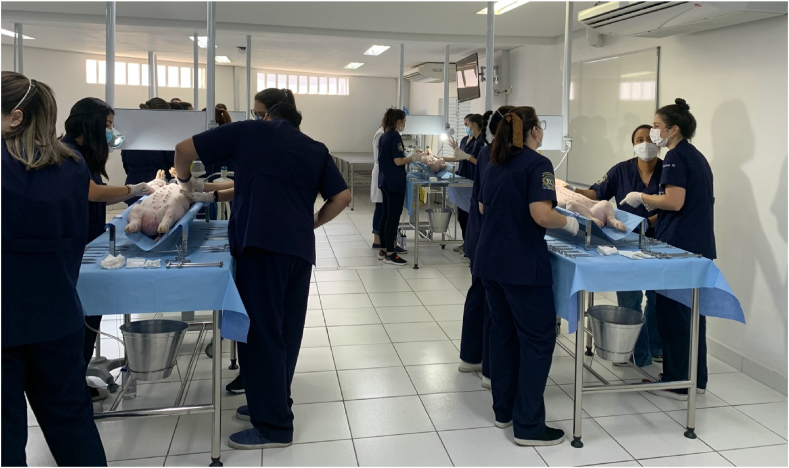
Surgical lab for animal practice – Using pigs for ATLS practical lesson: Chest drainage and Peritoneal lavage.

## 4 Discussion

In the literature, practical surgical teaching starts with sutures, which, from our perspective as well, seems reasonable. We believe that students need to learn how to handle surgical material and sutures before progressing to the actual surgical procedures [11].

Many different materials have been used to help students practice sutures. There is no material considered perfect for all sutures because of differences from the human skin. We chose ox tongue because it is easily accessible at a low cost in Brazil. Moreover, its superficial layer appropriately simulates aspects of the human skin, and its deep layer (muscle) mimics the human subcutaneous. In several studies, ox tongue was found to be the material that best met the requirements to simulate human skin and, as it is biodegradable, it can be discarded in the same way as other organic materials [[11], [12], [13], [14], [15]].

The natural progression after learning how to suture, from our perspective, is to perform tenorrhaphy and vascular anastomosis. In this way, the students understand concepts that will be used during animal surgeries and medical practice. Kessler tenorrhaphy training was performed first with cylindrical Styrofoam and then with a chicken foot. Vascular anastomosis was performed using a chicken neck, given its resemblance to human tissues and the possibility of performing both simple and continuous sutures for the anastomosis. The chicken trachea simulates arterial anastomosis, and the esophagus mimics venous anastomosis [16].

There are many advantages of using rabbits as models for surgical treatment, such as easy handling, affordable cost, low morbidity, no tendency toward hypoglycemia, no requirement for pre-operative fasting for the execution of abdominal surgical procedures, easy anatomy identification, and several similarities to the human anatomy, especially in relation to the digestive tract. Therefore, this model is considered appropriate for training procedures such as cystostomy, gastrostomy, appendectomy, and artery and vein dissection [[17], [18], [19]].

The main disadvantage of the rabbit model is related to endotracheal intubation, which is complicated due to the small size of the rabbit and its oral cavity shape. Considering this, we chose to include cricothyroidotomy and tracheostomy as part of the surgical training of the definitive airway. These were performed by students, always under direct supervision [19,20].

Pigs are the main animal species used for surgical training and are usually used as an alternative to dogs. The advantages of their use as a human tissue simulation model are the abdominal cavity with a basic muscular and fascial anatomy that is common to all mammals, and its abdomen may be compared in size to that of human adults. In this way, it is possible to simulate several types of procedures such as bowel resection with manual anastomosis, splenectomy, partial hepatectomy, and chest drainage. According to our schedule, students have the opportunity to perform chest drainage, orotracheal intubation, peritoneal lavage, and exploratory laparotomy. Few studies have reported the use of pigs for undergraduate medical training [[21], [22], [23], [24]].

Training with animal models has some benefits beyond the technical improvements. Some studies show that the surgical training performed in this way allows students to experience working together and develop the capacity to interact and work in synchrony with other students, helping them to work as a team. Other benefits noted are the improvement of students' confidence in their abilities and capacities as well as increased self-assessment. Thus, even with the high cost and other obstacles in the development of the animal model practice, the educational experience seems to make it all worth it, as this model can help to qualify future doctors for large centers as well as smaller cities where they would need to perform low-complexity surgical procedures [[23], [24], [25]].

## 5 Conclusion

Surgical training of medical undergraduates was demonstrated with an evolutionary intent, starting with simple sutures and ending up with basic emergency room surgical procedures.

## Funding sources

This research did not receive any specific grant from funding agencies in the public, commercial, or not-for-profit sectors.

## Declaration of competing interest

None.
