# Supplementary material for: How to do it: Teaching surgical skills to medical undergraduates
Source: Ann Med Surg (Lond). 2022 Sep 21;82:104617. doi: 10.1016/j.amsu.2022.104617 (PMC9577497; doi:10.1016/j.amsu.2022.104617)
Supplement: Multimedia component 1 [file mmc1.docx]

Our reference: <Annals of Medicine and Surgery>

P-copyright-v4/2021

**JOURNAL PUBLISHING AGREEMENT**

**PLEASE PROVIDE US WITH THE FOLLOWING INFORMATION, REVIEW OUR POLICIES AND THE JOURNAL PUBLISHING AGREEMENT, AND INDICATE YOUR ACCEPTANCE OF THE TERMS**

**YOUR STATUS (PLEASE MARK ALL THAT APPLY) I am the sole author of the manuscript**

Please indicate which of the below applies to you:

- I am a UK, Canadian or Australian Government employee and Crown Copyright is asserted
- I am a US Government employee and the Article is public domain and therefore the ‘Assignment of copyright’ clause does not apply
- I am a contractor of the US Government under contract number: ..........................................................................
- None of the above

**I am one author signing on behalf of all co-authors of the manuscript**

Please indicate which of the below applies to you:

- We are all US Government employees and the Article is public domain and therefore the ‘Assignment of copyright’ clause does not apply I am a US Government employee but some of my co-authors are not
- I am not a US Government employee but some of my co-authors are
- The work was performed by contractors of the US Government under contract number: ................................................
- All or some of the authors are UK, Canadian or Australian Government employees and Crown Copyright is asserted
- Some of the authors are employees of the UK, Canadian or Australian Government but Crown Copyright is not asserted
- None of the above

**The Article is a ‘work made for hire’ and I am signing as an authorized representative and on behalf of my employer**

Please indicate which of the below applies to you:

- The Article is authored by US Government employees and the Article is public domain and therefore the ‘Assignment of copyright’ clause does not apply
- The work was performed by contractors of the US Government under contract number: ...............................................
- The Article is authored by UK, Canadian or Australian Government employees and Crown Copyright is asserted
- None of the above

**Signed on Behalf of Corresponding Author**

Please enter a valid email address.
Please complete this section if you are not the corresponding author as listed above. A copy of the agreement will be sent to you and the corresponding author.

I am signing on behalf of the corresponding author
Name, job title and company (if employer representative) ........................................ E-mail address.......................................

Please tick the above boxes (as appropriate), review the Journal Publishing Agreement, and then sign and date the document in black ink.

Signed: ______________________________________

Name printed: _Victor Hugo Lara Cardoso de Sá__________________________________________
Title and Company (if employer representative): _________________________________________________________
Date: _______________________________________

Please return the completed and signed original of this form by mail or fax, or by e-mailing a scanned copy of the signed original, retaining a copy for your files, to:

Article entitled: <Item title>
Corresponding author: MD, MSC, MBA, Prof. Victor Hugo Lara Cardoso de Sá]

To be published in the journal: Annals of Medicine and Surgery

Our reference:

P-copyright-v4/2021

**THE JOURNAL PUBLISHING AGREEMENT**

Assignment of copyright
I hereby assign to the Copyright Owner the copyright in the manuscript identified above (where Crown Copyright is asserted, authors agree to grant an exclusive publishing and distribution license) and any tables, illustrations or other material submitted for publication as part of the manuscript (the “Article”). This assignment of rights means that I have granted to the Copyright Owner the exclusive right to publish and reproduce the Article, or any part of the Article, in print, electronic and all other media (whether now known or later developed), in any form, in all languages, throughout the world, for the full term of copyright, and the right to license others to do the same, effective when the Article is accepted for publication. This includes the right to enforce the rights granted hereunder against third parties.

Supplemental Materials
“Supplemental Materials” shall mean materials published as a supplemental part of the Article, including but not limited to graphical, illustrative, video and audio material.

With respect to any Supplemental Materials that I submit, the Copyright Owner shall have a perpetual worldwide non-exclusive right and license to publish, extract, reformat, adapt, build upon, index, redistribute, link to and otherwise use all or any part of the Supplemental Materials in all forms and media (whether now known or later developed), and to permit others to do so.

Research Data
“Research Data” shall mean the result of observations or experimentation that validate research findings and that are published separate to the Article, which can include but are not limited to raw data, processed data, software, algorithms, protocols, and methods.

With respect to any Research Data that I wish to make accessible on a site or through a service of the Copyright Owner, the Copyright Owner shall have a perpetual worldwide, non-exclusive right and license to publish, extract, reformat, index, adapt, build upon, redistribute, link to and otherwise use all or any part of the Research Data in all forms and media (whether now known or later developed), and to permit others to do so. Where I have selected a specific end user license under which the Research Data is to be made available on a site or through a service, the publisher shall apply that end user license to the Research Data on that site or service.

Reversion of rights
Articles may sometimes be accepted for publication but later rejected in the publication process, even in some cases after public posting in “Articles in Press” form, in which case all rights will revert to the author (see: https://www.elsevier.com/about/our-business/policies/article-withdrawal).

Revisions and addenda
I understand that no revisions, additional terms or addenda to this Journal Publishing Agreement can be accepted without the Copyright Owner’s express written consent. I understand that this Journal Publishing Agreement supersedes any previous agreements I have entered into with the Copyright Owner in relation to the Article from the date hereof.

Author Rights for Scholarly Purposes (see ‘Definitions’ clause below)
I understand that I retain or am hereby granted (without the need to obtain further permission) the Author Rights (see description below and definitions), and that no rights in patents, trademarks or other intellectual property rights are transferred to the Copyright Owner.

The Author Rights include the right to use the Preprint, Accepted Manuscript and the Published Journal Article for Personal Use, Internal Institutional Use. They also include the right to use these different versions of the Article for Scholarly Sharing purposes, which include sharing:

- the Preprint on any website or repository at any time;
- the Accepted Manuscript on certain websites and usually after an embargo period;
- the Published Journal Article only privately on certain websites, unless otherwise agreed by Copyright Owner.

In the case of the Accepted Manuscript and the Published Journal Article the Author Rights exclude Commercial Use (unless expressly agreed in writing by the Copyright Owner), other than use by the author in a subsequent compilation of the author’s works or to extend the Article to book length form or re-use by the author of portions or excerpts in other works (with full acknowledgment of the original publication of the Article).

Author Representations/Ethics and Disclosure/Sanctions
I affirm the Author Representations noted below, and confirm that I have reviewed and complied with the relevant Instructions to Authors, Ethics in Publishing policy, Declarations of Interest disclosure and information for authors from countries affected by sanctions (Iran, Cuba, Sudan, Burma, Syria, or Crimea). Please note that some journals may require that all co-authors sign and submit Declarations of Interest disclosure forms. I am also aware of the publisher’s policies with respect to retractions and withdrawal (https://www.elsevier.com/about/our-business/policies/article-withdrawal). For further information see the publishing ethics page at https://www.elsevier.com/about/our-business/policies/publishing-ethics and the journal home page. For further information on sanctions, see https://www.elsevier.com/about/our-business/policies/trade-sanctions.

*Author Representations*

- The Article I have submitted to the journal for review is original, has been written by the stated authors and has not been previously published.
- The Article was not submitted for review to another journal while under review by this journal and will not be submitted to any other journal.
- The Article and the Supplemental Materials do not infringe any copyright, violate any other intellectual property, privacy or other rights of

any person or entity, or contain any libellous or other unlawful matter.

- I have obtained written permission from copyright owners for any excerpts from copyrighted works that are included and have credited the

sources in the Article or the Supplemental Materials.

- Except as expressly set out in this Journal Publishing Agreement, the Article is not subject to any prior rights or licenses

Our reference: Annals of Medicine and Surgery P-copyright-v4/2021

- If I and/or any of my co-authors reside in Iran, Cuba, Sudan, Burma, Syria, or Crimea, the Article has been prepared in a personal, academic or research capacity and not as an official representative or otherwise on behalf of the relevant government or institution.
- If I am using any personal details or images of patients, research subjects or other individuals, I have obtained all consents required by applicable law and complied with the publisher’s policies relating to the use of such images or personal information. See https://www.elsevier.com/about/our-business/policies/patient-consent for further information.
- Any software contained in the Supplemental Materials is free from viruses, contaminants or worms.
- If the Article or any of the Supplemental Materials were prepared jointly with other authors, I have informed the co-author(s) of the terms of

this Journal Publishing Agreement and that I am signing on their behalf as their agent, and I am authorized to do so.

Governing Law and Jurisdiction

This Agreement will be governed by and construed in accordance with the laws of the country or state of the Copyright Owner (“the Governing State”), without regard to conflict of law principles, and the parties irrevocably consent to the exclusive jurisdiction of the courts of the Governing State.

For information on the publisher’s copyright and access policies, please see http://www.elsevier.com/copyright. **DEFINITIONS**

**Accepted Manuscript**

The manuscript of an Article that has been accepted for publication and which typically includes author-incorporated changes suggested during submission, peer review, and editor-author communications. The Accepted Manuscript should not be added to or enhanced in any way to appear more like, or to substitute for, the Published Journal Article. The Accepted Manuscript should include a link to the formal publication through the relevant DOI and should bear a Creative Commons CC BY-NC-ND license.

**Commercial Use**

The use or posting of Articles:

- for commercial gain – for example by associating advertising with the full-text of the Article, by providing hosting services to other

repositories or to other organizations, or charging fees for document delivery or access;

- to substitute for the services provided directly by the publisher – for example article aggregation, systematic distribution via e-mail lists or

share buttons, posting, indexing or linking for promotional/marketing activities by commercial companies for use by customers and/or intended target audience of such companies (e.g. pharmaceutical companies and healthcare professionals/physician-prescribers).

**Internal Institutional Use**

Use by the author's institution for classroom teaching at the institution and for internal training purposes (including distribution of copies, paper or electronic, and use in coursepacks and courseware programs, but not in MOOCs – Massive Open Online Courses) and inclusion of the Article in applications for grant funding or for patent applications. For authors employed by companies, the use by that company for internal training purposes.

**Personal Use**

Use by an author in the author’s classroom teaching (including distribution of copies, paper or electronic) or presentation by an author at a meeting or conference (including distribution of copies to the delegates attending such meeting), distribution of copies (including through e-mail) to known research colleagues for their personal use, use in a subsequent compilation of the author’s works, inclusion in a thesis or dissertation, preparation of other derivative works such as extending the Article to book-length form, or otherwise using or re-using portions or excerpts in other works (with full acknowledgment of the original publication of the Article).

**Preprint**

Author’s own write-up of research results and analysis that has not been peer reviewed, nor had any other value added to it by a publisher (such as formatting, copy editing, technical enhancements, and the like). Preprints should not be added to or enhanced in any way in order to appear more like, or to substitute for, the Published Journal Article.

**Published Journal Article**

The definitive final record of published research that appears or will appear in the journal and embodies all value-adding publisher activities including peer review co-ordination, copy-editing, formatting, (if relevant) pagination, and online enrichment.

**Scholarly Sharing**

***Preprint***: Sharing of Preprints by an author on any website or repository at any time. When the Article is accepted, the author is encouraged to include a link to the formal publication through the relevant DOI. The author can also update the Preprint on arXiv or RePEc with the Accepted Manuscript.

***Accepted Manuscript:***

**(i)**

• •

•

**immediately on acceptance**: sharing of the Accepted Manuscript by an author:
via the author’s non-commercial personal homepage or blog
via the author’s research institute or institutional repository for Internal Institutional Use or as part of an invitation-only research collaboration work-group
directly by providing copies to the author’s students or to research collaborators for their personal use

Our reference: Annals of Medicine and Surgery P-copyright-v4/2021

• for private scholarly sharing as part of an invitation-only work group on commercial sites with which the publisher has a hosting

agreement

**(ii) after the embargo period:** an author may share the Accepted Manuscript via non-commercial hosting platforms (such as the author’s institutional repository) and via commercial sites with which the publisher has a hosting agreement.

To check the embargo period for the journal, go to http://www.elsevier.com/embargoperiodlist

The publisher has agreements with certain funding agencies that may permit shorter embargo periods and/or different sharing guidelines. To learn more about the publisher's policies and agreements with such agencies or institutions go to http://www.elsevier.com/fundingbodyagreements.

***Published Journal Article:*** the author may share a link to the formal publication through the relevant DOI or may share the Published Journal Article privately with students or colleagues for their personal use, or privately as part of an invitation-only work group on commercial sites with which the publisher has a hosting agreement. Additionally theses and dissertations which contain embedded Published Journal Articles as part of the formal submission may be hosted publicly by the awarding institution with a link to the formal publication through the relevant DOI. Any other sharing of Published Journal Articles is by agreement with the publisher only.

For more information on the publisher’s sharing policies please see https://www.elsevier.com/sharingpolicy.
